# Presbycusis and the Aging of Eye Movement: Common Attention Mechanisms

**DOI:** 10.3390/brainsci12010107

**Published:** 2022-01-13

**Authors:** Martin Chavant, Zoï Kapoula

**Affiliations:** IRIS Laboratory, Neurophysiology of Binocular Motor Control and Vision, CNRS UAR 2022, University of Paris, 45 Rue des Saints Pères, 75006 Paris, France; martin.chavant@etu.u-paris.fr

**Keywords:** presbycusis, saccade and vergence latency, attention, aging

## Abstract

Presbycusis, physiological age-related hearing loss, is a major health problem because it is the most common cause of hearing impairment, and its impact will grow in the coming years with the aging population. Besides auditory consequences, the literature recently found an association between hearing loss and cognitive decline over the last two decades, emphasizing the importance of the early detection of presbycusis. However, the current hearing tests are not sufficient to detect presbycusis in some cases. Furthermore, the underlying mechanisms of this association are still under discussion, calling for a new field of research on that topic. In that context, this study investigates for the first time the interaction between presbycusis, eye movement latency and Stroop scores for a normal aging population. Hearing abilities, eye movement latency and the Stroop Victoria test were measured for 69 elderly (mean 66.7 ± 8.4) and 30 young (mean 25.3 ± 2.7) participants. The results indicated a significant relationship between saccade latency and speech audiometry in the silence score, independently from age. These promising results suggest common attentional mechanisms between speech processing and saccade latency. The results are discussed regarding the relationship between hearing and cognition, and regarding the perspective of expanding new tools for presbycusis diagnosis.

## 1. Introduction

Aging in the brain involves a loss of sensory processing, motor performance and cognitive function caused by a loss of synaptic contact [1].

The physiological loss of hearing with age, presbycusis, is a well-known phenomenon. This is the most common cause of hearing impairment, and is caused by multiple intrinsic and extrinsic risk factors (noise exposure, genetic predisposition, and health comorbidity). The prevalence of hearing loss for the senior population varies depending on studies, as studies use different hearing variables, different cutoffs for the definition of hearing loss, and different demographic characteristics in their cohorts [2]. A study on 717 US adults above 70 years of age found a hearing loss prevalence of 63.1% (hearing loss was defined when the average pure tone detection threshold across 500, 1 k, 2 k and 4 k was above 25 dB HL for the best ear) [3].

The auditory consequences of presbycusis are a bilateral and symmetric increase of the hearing threshold (beginning with the high frequencies), a decrease of frequency resolution, and a loss of comprehension, especially in noisy environments [4,5,6]. However, other consequences, which are less obvious, exist. Studies from the last decade have highlighted the relationship between presbycusis and cognition [7]. Indeed, they have shown that hearing loss is related to an accelerating cognitive decline and an increased risk of dementia [8,9,10]. A recent study even found that presbycusis is the primary risk factor of dementia in middle life that could be controlled [11]. The underlying mechanisms of this association remain vague, and are currently being discussed in the literature. Hearing loss and cognitive decline may arise from a common etiology, such as age-related vascular risk factors or neurodegenerative mechanisms. Furthermore, hearing loss can directly be linked to cognitive health by reducing social engagement [12], overloading cognitive resources [13], changing brain structure and function [14,15,16,17,18], or deteriorating the auditory/cognitive system [8]. It is likely that all of these explanations are not exclusive, and each may contribute to this association between hearing loss and cognitive decline.

The detection of presbycusis is currently performed with three main hearing tests: pure-tone audiometry, and speech intelligibility in silence and in noise. However, these hearing tests could sometimes fail to detect subtle deficiencies of hearing capacities [19]. Normal hearing thresholds do not necessarily imply the absence of hearing issues, as some degraded hearing properties can lead to poorer hearing processing without hearing thresholds’ elevation. Similarly, good performance in speech intelligibility tasks does not reflect normal hearing. Indeed, these tests performed in a sound booth cabin are not ecological, i.e., they cannot mimic all of the situations encountered in real life, and therefore do not provide such hearing loss indicators as the listening effort or the mental replacement.

In that context, the present study focuses on two aspects concerning the association between hearing loss and cognitive decline. The first one concerns the need to understand the multisensory mechanisms underpinning the ways in which hearing loss and cognitive decline relate to each other. The second, more translational aspect, aims to provide new sensitive tools that can contribute to the detection of even mild hearing deterioration as early as possible in elderly patients.

These problems will be treated by assessing the relationship between presbycusis and eye movement latency.

Eye movements are an ideal tool to examine the perception and action mechanisms of the brain, such as cognition, motor control and memory [20]. Indeed, their dynamic properties and neurobiological substrate are well-known, and their measurements are easy to perform. The current study will focus on two specific eye movements: saccade, allowing lateral movement, and vergence allowing movement in depth. Saccades are divided into left and right saccade. Vergences are divided into convergence, to look at an object that gets closer, and divergence to look at an object that goes further away.

The eye movement latency is the reaction time to initiate the eye movement to reach a target. In a more general way, the reaction time is related to executive cognitive function [21,22,23]. The eye movement latency process is comprised of several steps: the release of ocular fixation, the shift of visual attention, the computation of the eye movement metrics, and the decision to move the eyes. The cortical areas, including the frontal and parietal cortices, participate in this process. Age negatively impacts eye movement latency in later life. More precisely, during the lifespan, there is at first a reduction of saccade latency between childhood and adulthood [24,25,26], then a period of stabilization until around 50 years, finishing with an increase at older ages [26,27,28,29,30]. The literature for vergence eye movement is scarce, but as for saccade latency, it shows an increase of latency with age [31,32].

The relationship between eye movement and cognitive executive functions is rather restricted to studies on saccade latency. For instance, the saccades latency is longer for people with mild cognitive impairment than for healthy elderly people, and a significant correlation between saccade latency and the MMSE (the Mini Mental State Evaluation) has been reported [33]. Others found a significant increase in saccade latency for people with Alzheimer’s disease compared to healthy individuals [34,35].

The fact that hearing and eye movement reaction times are both related to cognition suggests a potential relationship between them. This study aims to open up a new research avenue on the triple relationship between age-related hearing loss, saccade and vergence eye movement, and cognition. The results could bring new insights for (i) the objectification and the understanding of the association between hearing loss and cognitive decline, and (ii) the development of new clinical tools for the diagnosis of presbycusis.

To do this, we will assess the interaction between three functions: presbycusis, saccade and vergence latency, and executive cognitive function. The cognitive test used here will be the Stroop test, a golden test to measure the selective attention and inhibition capacities [36], which are also damaged by age [37,38,39,40,41].

We hypothesize that poorer hearing will be related to longer latencies, and perhaps to lower scores for cognitive executive functions.

The results confirm a link between eye movement and hearing loss, and are of both theoretical and clinical interest.

## 2. Materials and Methods

### 2.1. Participants

The participants were divided into two groups: an elderly group (Group E) composed of 69 participants aged between 51 and 84 years (mean 66.7 +8.4, 18 M and 51 W), and a young group (Group Y) composed of 30 participants aged between 21 and 30 years (mean 25.3 ± 2.68, 17 M and 13 W). Group E was recruited by the RISC (*relai d’information des sciences cognitives*, France) platform of the CNRS, or by contacting associations which were likely to have people of appropriate ages. Some of them were retired, while others were still working. All of the participants were autonomous, and came to the laboratory without assistance. We can consider this sample as an average elderly population. Group Y was composed of people working in the same building.

All of these participants had good sight or wore visual correction. None of the participants showed neurological or psychiatric disorders, or received any medication that could affect their sensory and motor functions. Finally, none had auditory or oculomotor pathologies. Among Group E participants, 5% of them were being treated for diabetes, 17% were being treated for blood pressure issues, none had renal failure, and 14% had vascular issues (60% of which were treated).

Informed consent was obtained from all of the participants after the nature of the procedure had been explained.

Rather than focusing on the elderly group, which became smaller than planned due to the COVID 19 pandemic, we sought to add a group of young participants, enabling us to evaluate aging relative to the performances of young people, as well as progressive aging within the elderly group itself.

The study was conducted in accordance with the Declaration of Helsinki, and approved by Ethics Committee “Ile de France II” (N° ID RCB: 019-A02602-55, approved the 10/03/2020). 

### 2.2. Hearing Tests

An audiometrist assessed all of the hearing tests with an audiometer of the brand Interacoustics (model AD629) in a sound booth calibrated cabin. These tests were composed of pure-tone hearing threshold audiometry and two speech recognition tests, one in silence and one in noise. An otoscopic evaluation was first performed to detect any foreign body in the outer ear canal that could bias the audiometric results.

Pure-tone hearing threshold audiometry (also known as tonal audiometry) measures audibility, e.g., the minimum intensity required to detect a sound. It was realized with a headset (audiometric TDH-39P), with one ear tested at the time. The score extracted from the pure-tone hearing threshold audiometry is the best ear’s PTA (pure-tone average). The hearing thresholds in dB HL for pure-tones of 250, 500, 750, 1000, 2000, 3000 and 4000 Hz were determined with 5-dB steps. Then, the PTA was calculated by meaning all of these thresholds. We decided to keep the PTA of the better ear in order to follow the hearing loss definition of the world health organization (WHO) [42], which is when the better PTA of the two ears is above 20 dB HL.

**Figure 1:**
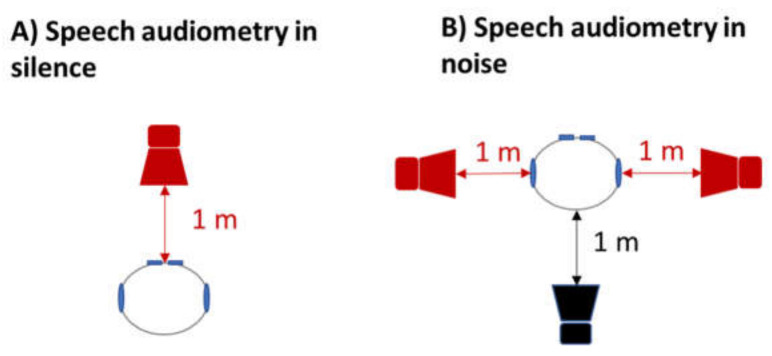
Top view of the position of the speakers for (**A**) the speech audiometry in silence test and (**B**) the speech audiometry in noise test. The red speakers are for the speech signal. The black speaker is for the noise signal.

The speech audiometry in silence was realized with a loudspeaker situated at 1 m in front of the participant (azimuth 90°, Figure 1A). From this loudspeaker (brand Tangent, model EVO) were sent different lists of words with different step intensity levels: either 70, 60, 50, 40, 30, 20 or 10 dB SPL. The lists were the Lafon cochlear lists, which are composed of 17 monosyllabic words of 3 phonemes (51 phonemes) [43]. Each list was assigned a score of comprehension, representing the percentage of phonemes correctly repeated, out of the 51 phonemes of a list. The intensity level of the first list was chosen concerning the PTA scores (assessed just beforehand) to be well-heard by the participant. Each following list was then sent with a lower step intensity. The score extracted from the speech audiometry in silence was the SRT50 (speech recognition threshold 50%), representing the intensity required in dB SPL (sound pressure level) such that the participant repeats 50% of the phonemes. In this study, the SRT50 was estimated by a cross-product between the intensity needed to obtain a score above 50% and the intensity needed to obtain a score under 50%.

The speech audiometry in noise was realized with three loudspeakers (brand Tangent, model EVO) situated at 1 m from the participant (Figure 1B): one behind him (azimuth 270°), one to his right (azimuth 180°) and one to his left (azimuth 0°). From the two loudspeakers on the right and left were sent the Lafon cochlear lists (the same speech signal as for the speech audiometry in silence). From the loudspeaker situated behind the participant was sent a noise signal called the OVG (*Onde Vocale Globale* in French) [44]. This noise is composed of a mix of two couples, one French and one English, speaking simultaneously, resulting in incomprehensible babble noise. Similarly to the audiometry in silence, each Lafon cochlear list was assigned a score of comprehension, representing the percentage of phonemes correctly repeated, out of the 51 phonemes. The score extracted from the speech audiometry in noise was based on the Signal-to-noise ratio (SNR). The SNR represents the extent to which the speech signal is higher or lower in intensity than the noise intensity. It is calculated by deducting the intensities in dB SPL of the speech list and the noise (SNR = signal intensity − noise intensity). The SNR was varied for each new Lafon cochlear list by changing the noise intensity while the intensity of Lafon cochlear lists remained unaltered. Thus, during all of their speech audiometry in noise, each participant had a specific unchanged intensity for all of their Lafon cochlear lists. For each participant, the intensity of the Lafon cochlear lists was chosen by taking the lower intensity in the speech audiometry in silence giving the best score (example: if in the speech-in-silence test, participant A had a recognition score of 100% for the list at 60 dB SPL, 100% for the list at 50 dB SPL and 82% for the list at 40 dB SPL, then the intensity of the lists for the whole speech audiometry in noise test would be set at 50 dB SPL). The first Lafon cochlear list was sent with a SNR at 0 (speech and noise at the same intensity level). We then decreased the SNR by 5 for each list (by increasing the noise level by steps of 5 dB SPL). The variable extracted from the speech-in-noise test was the SNR50 (signal-to-noise ratio 50%), representing the SNR required to obtain a phoneme discrimination score of 50%. As for the speech comprehension in silence test, the SNR50 was estimated by a cross-product between the SNR needed to obtain a score above 50% and the SNR needed to obtain a score under 50%. Consequently, this test assessed the degradation of comprehension by the noise for a signal completely understood in silence.

In total, 62 participants of Group E (62/69, 89.9%) and 19 participants of Group Y (19/30, 63.3%) performed the hearing tests; note that the hearing tests were carried out in a different place than the eye movement tests, and many of the young participants were no longer available.

### 2.3. Oculomotor Tests

The different oculomotor movements (divergence, convergence, left saccade and right saccade) were tested via the REMOBI device, as first described by Kapoula et al. [45].

**Figure 2:**
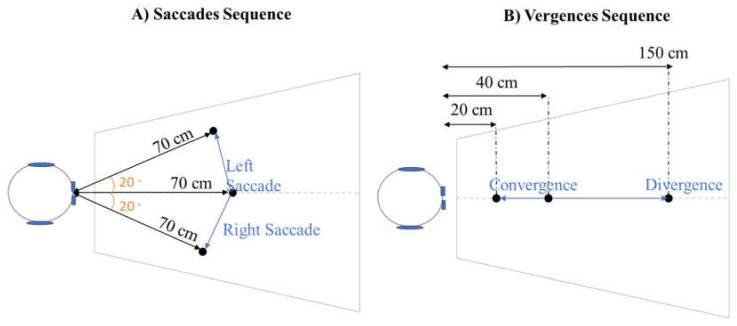
Saccade and vergence sequences on the REMOBI device. (**A**) Top-view of the position of the LEDs for the saccade sequence. (**B**) Top-view of the position of the LEDs for the vergence sequence.

The REMOBI device is a visio-acoustic surface composed of 48 LEDs (with a nominal frequency of 626 nm, intensity 180 mCd, and a diameter of 3 mm) embedded at 4 isovergence arcs. The device includes different sequences, lighting up the LEDs in different patterns. The participants sat in front of the REMOBI device, which was placed at eye level, and were instructed to fixate the activated LED as quickly and accurately as possible and maintain the fixation. The sequence chosen on the REMOBI device enables testing a specific kind of eye movement. Two sequences were used in this study: the saccade sequence, measuring the left and right saccades, and the vergence sequence, measuring divergence and convergence (Figure 2).

The saccade sequence comprises 20 trials of right saccades and 20 trials of left saccades, randomly interleaved. During each trial, a central LED, situated at 70 cm in front of the participant (the same distance from his left and right eye), is switched on at a random time between 1200 and 1800 ms. Then, a lateral LED to the right or to the left is lit for 2000 ms, following an overlap paradigm, i.e., the central LED switches off 200 ms after the onset of the lateral LED. The lateral LED forms an angle of 20° with the central LED, calling for a left saccade or right saccade of 20°.

The vergence sequence comprises 20 trials of divergence and 20 trials of convergence, randomly interleaved. During each trial, a central LED—situated 40 cm in front of the participant (the same distance from his left and right eye)—is switched on at a random time between 1200 and 1800 ms. Then a nearest LED or a farthest LED is lit for 2000 ms, following an overlap paradigm. These two LEDs are on the same axis as the central LED (the same distance from the left and right eyes of the participant). The nearest LED is situated 20 cm from the participant, calling for a convergence angle of 8.76°. The farthest LED is situated 150 cm from the participant, calling for a divergence angle of 6.5°.

Between the trials for the saccade and vergence sequences, a blanked period of 300 ms to 700 ms was applied. All of these values are given with a pupillary distance of 62 mm.

The eye movements were recorded binocularly with the head-mounted video-oculography device, Pupil Core (Pupil Labs, 12047 Berlin, Germany).

### 2.4. Eye Movement Analysis

The data recorded with the Pupil Labs eye tracker was analyzed with the AIDEAL software (pending international patent application: PCT/EP2021/062224 7 May 2021). The signal was derived by calculating the difference between the two eyes from the individual calibrated eye position signals (i.e., left eye-right eye). The onset and the offset of the saccades were defined as the moments where the velocity went above or below 10% of the peak velocity. The onset and the offset of the vergences were defined as the moments where the velocity went above or below 5°/s. These criteria are standard, and were applied automatically by the AIDEAL software. Trials with blinks were excluded.

**Figure 3:**
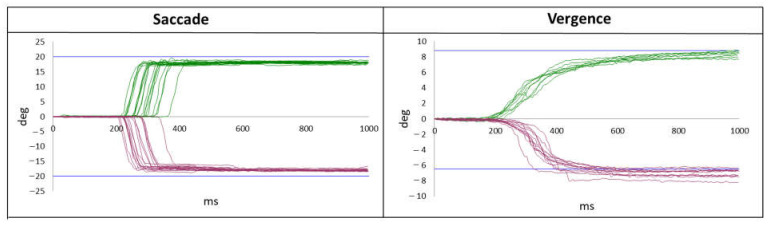
The kind of results given by the AIDEAL software, permitting a quick overview of the results for vergence and the saccade sequence with time in ms on the X-axis and degrees on the Y-axis. The green curves represent the convergence and right saccades. The purple curves represent divergence and left saccades. Each curve represents one trial.

The results are given in an excel spreadsheet and graphs (Figure 3).

### 2.5. Stroop Tests

The Stroop test is a cognitive test assessing executive functions such as selective attention or inhibition capacities. It consists of orally enumerating the font colors of a list of words with a different meaning than their color (ex: the word “blue” printed in red).

The brain has to inhibit the information given by the word’s meaning, which is the most protruding and intuitive, to focus on the information given by the printed colors. In other words, it has to focus on specific information while ignoring other information.

The Stroop test was first described in 1935 by J.R. Stroop [36]. Many variations of this original test were created, but they followed the same principle. The different Stroop tests are always composed of three or four parts, from simple tasks such as reading words printed in black or the color recognition of colored dots, to the final and more complex task, cited above, of color enumeration with incongruent words.

In this article, the selected version of the Stroop test is the French Stroop Victoria [46]. This version of the Stroop test was chosen because of its short administration time, being appropriate for usage in an elderly population, and the provision of a normative database on 244 healthy community-dwelling adults living in Montpellier and Lille (mean age 65.83 SD = 10.71).

In this version, the participant has to list the color of 24 items as quickly as possible (6 lines of 4 items) in three different conditions. The possible colors of the items are blue, green, yellow or red. The first condition is the “Dot” condition, where the items are dots. The second condition is the “Word” condition, where the items are the French words “*mais”* (but), “*pour”* (for), “*donc”* (thus) and “*quand”* (when). The third condition is the “Interference” condition, where the items are the words “*bleu”* (blue), “*vert”* (green), “*jaune”* (yellow) and “*rouge”* (red). The words in this last condition are incongruent, i.e., the color ink of the word is not the same as the word’s signification (example: the word “*rouge”* (red) printed in green).

In this article, Stroop_D represents the time to perform the “Dot” condition, Stroop_W represents the time to perform the “Word” condition, and Stroop_I represents the time to perform the “Interference” condition. From these variables are also calculated the Stroop_I/D, which represents the ratio of Stroop_I over Stroop_D; and Stroop_W/D, which represents the ratio of Stroop_W over Stroop_D.

Thus, the Stroop Victoria first assesses the speed of color denomination (Stroop_D). Then, it assesses the same variable but in the presence of distracting information, i.e., the meaning of the words (Stroop_W and Stroop_I). The ratios Stroop_W/D and Stroop_I/D represent the behavioral impact of this distracting information on the speed of color denomination. The difference between Stroop_W/D and Stroop_I/D is about the strength of their interference effect. The distracting information given by the “Interference” condition is stronger than that given by the “Word” condition. Thus, the Stroop_W/D assesses the behavioral impact of a weak interference, while the Stroop_I/D assesses the behavioral impact of a strong interference.

Besides this, the study of aging’s effect on inhibition and selective attention will be more specific with Stroop_I/D than with Stroop_I. Indeed, if the increase of Stroop_I with age could reflect the loss of selective attention capacities, it can also reflect a general slowing due to age (in this last case, Stroop_D will be increased too). The age-related general slowing is a robust finding in studies. This behavior slowing appears for motor responses and sensory processes, and becomes more important with complex tasks [47,48]. The calculation of this ratio variable (Stroop_I/D) reduces the influence of age-related general slowing [49].

### 2.6. Data Analyses

Aging’s effects on hearing, eye movement latency and Stroop scores are measured with simple linear regressions and correlations: Hearing ~ Age, Latency ~ Age, and Stroop ~ Age. These results are presented in the Section 3.3—Aging Effects.

The relationships between hearing, eye movement latency and Stroop scores are investigated two by two: hearing VS eye movement latency, hearing VS Stroop scores and latency VS Stroop scores. As all of these parameters deteriorate with age, the results may be skewed by their confounding effects. In order to avoid this, the simple linear regression will be abandoned in favor of multiple regression analysis, adding age as an explanatory variable: Hearing ~ Latency + Age, Hearing ~ Stroop + Age, and Latency ~ Stroop + Age. These results are presented in the Section 3.4—Links between Eye Movement Latency, Hearing and Selective Attention, Independently of Age. 

## 3. Results

The results are organized as follows: (i) characteristics of the population in terms of hearing and cognition relative to healthy standards; (ii) links between the three hearing scores via Pearson correlation; (iii) aging’s effect on the hearing, eye movement latency and Stroop scores via Pearson correlation; (iv) links between hearing, eye movement latency and Stroop scores via multiple regression analysis.

### 3.1. Characterization of Group E

**Figure 4:**
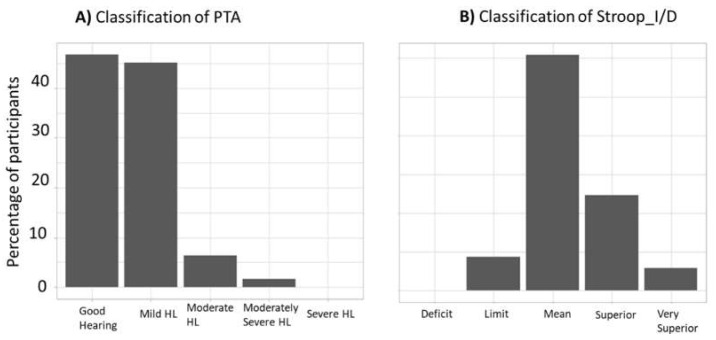
Hearing loss (HL) and Stroop score characterization of Group E. (**A**) Classification of the PTA according to the WHO scale, for Group E. (**B**) Classification of the Stroop_I/D according to the model built in the study of Bayard et al. [34], for Group E. This model allows the categorization of the Stroop_I/D score in the function of the participant’s age above 50 years. The score can be classified into five categories: “deficit”, “limit”, “mean”, “superior”, and “very superior”.

The classification of hearing loss and the Stroop results for Group E are shown in Figure 4. The hearing loss classification is according to the WHO scale of hearing loss [42], and Stroop result classification is according to the model built in the study of Bayard et al. [46]. This characterization was made to assess whether Group E was in the normal standards of aging.

According to the WHO hearing loss scale [30], Figure 4A shows that for the 62 participants of Group E who passed the hearing tests, 46% had normal hearing, 45% presented mild hearing loss (PTA of the better ear between 20 and 35 dB HL), 6% presented moderate hearing loss (PTA of the better ear between 35 and 50 dB HL), and 1% presented moderately severe hearing loss (PTA of the better ear between 50 and 65 dB HL).

These results, discussed below, are in the normal standard for hearing aging. Of the 19 participants of Group Y who passed the hearing tests, none of them had a PTA superior to 20 dB HL or were considered to have hearing issues. Figure 4B shows that, given the classification provided by the French Stroop Victoria test, none of the participants of Group E were classifiable as presenting cognitive deficiency. Indeed, none of the Stroop_I/D scores were in the “deficit” category, 9% of them were in the “limit” category, 60% were in the “mean” category, 25% were in the “superior” category, and 6% were in the “very superior” category.

To sum up, the hearing and Stroop scores pointed toward a healthy aging population.

### 3.2. Correlation between the Hearing Tests

**Figure 5:**
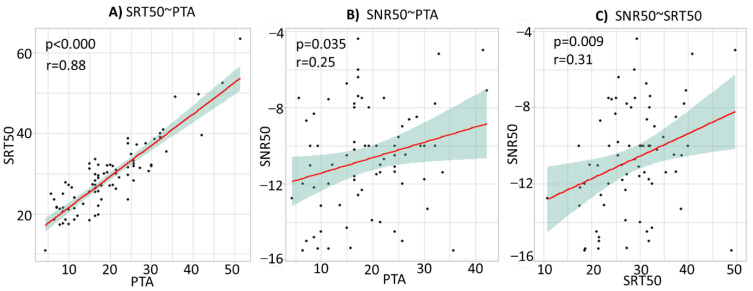
Correlations between the different hearing tests (pure-tone hearing threshold, speech-in-silence and speech-in-noise), for the whole population (Group Y + Group E). (**A**) Correlation and regression line between the pure-tone hearing threshold (PTA) and the speech-in-silence score (SRT50). (**B**) Correlation and regression line between the pure-tone hearing threshold (PTA) and speech-in-noise score (SNR50). (**C**) Correlation and regression line between the speech-in-silence (SRT50) and speech-in-noise (SNR50) scores. The red lines represent the regression lines. The grey areas represent the 95% confidence level interval. The “*r*” represents the Pearson correlation coefficients, and the “*p*” represents the significances of the slopes of the regression lines.

The results assessing the correlations and regression lines between the different hearing tests for the whole population (Group Y + Group E) are in Figure 5.

The correlations in Figure 5A indicate a strong correlation between the PTA and the SRT50 (*r* = 0.88, *p* = 0.000). However, the results in Figure 5B,C show that the other correlations, e.g., PTA vs SNR50 (*r* = 0.25, *p* = 0.035), or SNR50 vs SRT50 (*r* = 0.31, *p* = 0.009) are weaker, albeit statistically significant. These results indicate the difficulty of predicting speech-in-noise ability based on pure-tone threshold audiometry.

### 3.3. Aging Effects

Before assessing the interaction between hearing, eye movement latency and inhibition capacities, it is important to analyze their interaction with age. Age is a preponderant factor in this study, and it is known that it affects both of them. This analysis enables us to check whether or not the population is aging normally.

#### 3.3.1. On Hearing

**Figure 6:**
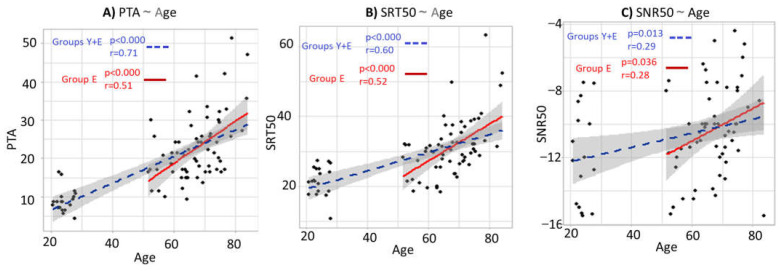
Correlations between hearing and age for the whole population (Group Y + Group E) and Group E only. (**A**) Correlation and regression line between the pure-tone hearing threshold (PTA) and age. (**B**) Correlation and regression line between the speech-in-silence score (SRT50) and age. (**C**) Correlation and regression line between the speech-in-noise score (SNR50) and age. The blue dashed lines represent the regression lines for the whole population (Group Y + Group E). The red lines represent the regression lines for Group E only. The grey areas represent the 95% confidence level interval. The “*r*” represents the Pearson correlation coefficients, and the “*p*” represents the significances of the slopes of the regression lines.

The results assessing the correlations and regression lines between the different hearing tests and age for the whole population (Group Y + Group E) and Group E are in Figure 6.

The results highlight the global deterioration of hearing capacities across the lifespan. The slopes of the regression lines for the whole population (blue dashed lines) are positive and statistically significant for the audibility (Figure 6A), speech-in-silence perception (Figure 6B) and speech-in-noise perception (Figure 6C), showing a loss of performance between the young and the elderly population. Moreover, these trends remain when only considering the elderly group, as shown by the significant slopes of the red regression lines. Thus, the hearing capacities continue to decrease within the elderly population. The correlation between SNR50 and age in Figure 6C shows that the variability is more important regarding the speech-in-noise capacities, whether this is concerning the young population or the elderly population. This last result brings additional evidence for the need to assess speech-in-noise abilities, even for young subjects with normal scores in the pure-tone hearing threshold audiometry.

#### 3.3.2. On Eye Movement Latency

**Figure 7:**
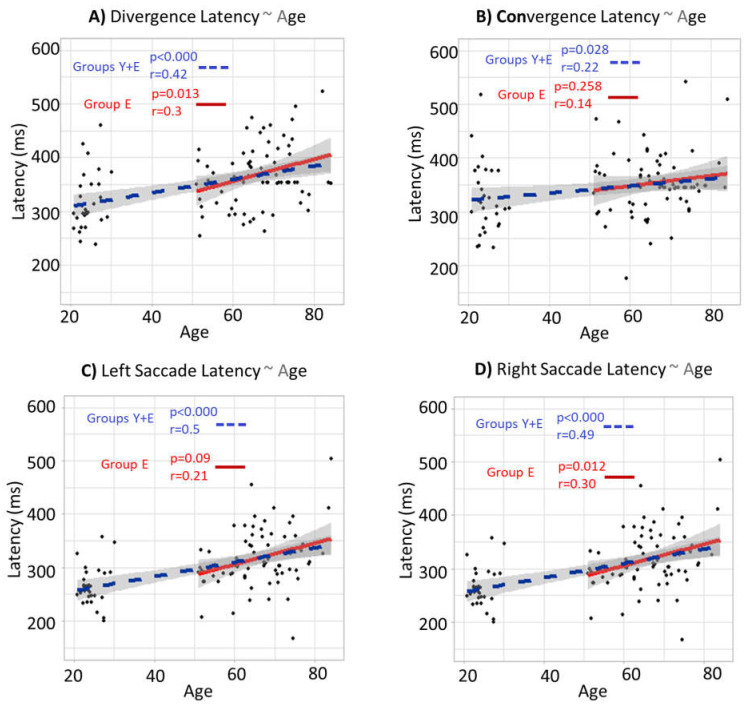
Correlations between eye movement latency and age for the whole population (Group Y + Group E) and Group E only. (**A**) Correlation and regression line between the divergence latency and age. (**B**) Correlation and regression line between the convergence latency and age. (**C**) Correlation and regression line between the left saccade latency (SNR50) and age. (**D**) Correlation and regression line between the right saccade latency and age. The blue dashed lines represent the regression lines for the whole population (Group Y + Group E). The red lines represent the regression lines for Group E only. The grey areas represent the 95% confidence level interval. The “*r*” represents the Pearson correlation coefficients, and the “*p*” represents the significances of the slopes of the regression lines.

The results assessing the correlations and regression lines between the different eye movement latencies and age for the whole population (Group Y + Group E) and Group E are in Figure 7.

The results in Figure 7 highlight the global increase of eye movement reaction time across the lifespan. The significant slopes of the regression line for the whole population (blue dashed lines) show an increase of the latency between the young and elderly population for divergence (Figure 7A), convergence (Figure 7B), left saccade (Figure 7C) and right saccade (Figure 7D). Additionally, the blue regression lines for the elderly group indicate that this increase of the latency of eye movements with age continues for an elderly population, except for the convergence.

#### 3.3.3. On Selective Attention (Stroop Test)

**Figure 8:**
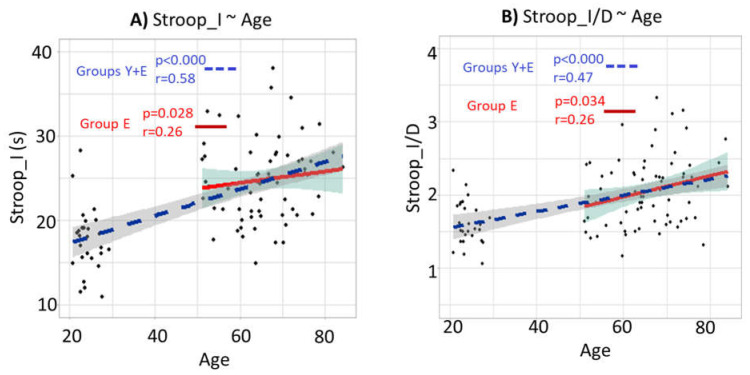
Correlations between Stroop scores and age for the whole population (Group Y + Group E) and Group E only. (**A**) Correlation and regression line between Stroop_I and age. (**B**) Correlation and regression line between Stroop_I/D and age. The blue dashed lines represent the regression lines for the whole population (Group Y + Group E). The red lines represent the regression lines for Group E only. The grey areas represent the 95% confidence level interval. The “*r*” represents the Pearson correlation coefficients, and the “*p*” represents the significances of the slopes of the regression lines.

The results assessing the correlations and regression lines between the different Stroop scores and age for the whole population (Group Y + Group E) and Group E are in Figure 8 and Table 1.

The results of the linear regressions and the correlation of the different Stroop scores as a function of age are shown in Table 1. The column “a” gives the slopes of the regression line, and their significances are indicated with asterisks: “***” for a *p* inferior to 0.001, “**” for a *p* between 0.001 and 0.01, “*” for a *p* between 0.05 and 0.01, and “.” for a *p* between 0.1 and 0.05. The column “cor” gives the Pearson correlation values.

Regarding the results for the whole population (Group Y + Group E, in the left part of Table 1), the slopes of the regression lines are positive and significant for all of the Stroop scores. The color-enumerating capacity measured with the “Dot” condition is a basic cognitive skill, and can be assimilated to the reading ability. The increased time for the elderly relative to the young is presumably due to the age-related general slowing [35,36]. Thus, these results confirm the age-related general slowing and the decrease of inhibition capacity in elderly persons.

Regarding the results for Group E only (in the right part of Table 1), the slopes of the regression lines are positive and significant for all of the Stroop scores except for Stroop_D, suggesting that the loss of inhibition capacities continues inside an elderly group.

To sum up, the ensemble of our tests confirms a normally aging population: all of the scores of Group E are worse than those of Group Y, as expected, and are within the normal range for their age rank. Now that this characterization is complete, we will assess the main point of this study: the triple relationship between age-related hearing loss, saccade and vergence eye movement latency, and cognition.

### 3.4. Links between Eye Movement Latency, Hearing and Selective Attention, Independently of Age

The following analyses aim to evaluate the relationships between latency, hearing and Stroop scores.

#### 3.4.1. Hearing and Eye Movement Latency

This part presents the results of different multiple regressions analyses, all assessing the effect of eye movement latency and age on hearing. Table 2 aggregates all of these results for the whole population (Group Y + Group E). The first row assesses the effect of eye movement latency and age on PTA (PTA ~ Latency + Age), the second row on SRT50 (SRT50 ~ Latency + Age), and the third row on SNR50 (SNR50 ~ Latency + Age). For each row, the first line shows the effect of latency on the hearing variable, independent of age. The second line shows the effect of age on the hearing variable, independent of latency. The columns indicate the eye movement tested (divergence, convergence, left and right saccade). Thus, for example, the first row and first column result from the multiple regression PTA ~ Divergence Latency + Age. The values to focus on are the “a”, representing the slope of the regression line. Their significance level is indicated with asterisks: “***” for a *p* inferior to 0.001, “**” for a *p* between 0.001 and 0.01, “*” for a *p* between 0.05 and 0.01, and “.” for a *p* between 0.1 and 0.05. Table 3 and Table 4 are structured similarly 

The results show a significant relationship between SRT50 and saccade latency independently from age. Looking at the third and fourth column and second line, the slopes of the regression lines for the left and right saccades are significant and positive. They indicate that the SRT50 of the left saccade increases by 0.033 dB SPL, and the SRT50 of the right saccade increases by 0.043 when the latency increases by 1 ms. In other words, the speech comprehension decrease is associated with the increase of the reaction time to initiate saccades. This effect remains when focusing on Group E only.

Thus, these results suggest that saccade latency may be a tool to target the cognitive consequences of presbycusis. This idea is discussed later.

#### 3.4.2. Hearing and Stroop Scores

This part presents the results of different multiple regressions analyses, all assessing the effect of Stroop scores and age on hearing. Table 3 aggregates all of these results for the whole population (Group Y + Group E). The way to read it follows the same instructions as for Table 2, described in the Section 3.4.1—Hearing and Eye Movement Latency. None of the slopes of regression lines (the “a” values) assessing the association between the Stroop scores and hearing scores are significant, which is also the case when considering Group E alone. Thus, although the hearing and Stroop scores are both affected by age similarly, they do not affect each other. These results suggest that the visual Stroop is not an adequate test to assess a potential hearing-related decline of cognition.

#### 3.4.3. Eye Movement Latency and Stroop Scores

This part presents the results of different multiple regressions analyses, all assessing the effect of Stroop scores and eye movement latency. Table 4 aggregates all of these results for the whole population (Group Y + Group E). The way to read it follows the same instructions as for Table 2, described in the Section 3.4.1—Hearing and Eye Movement Latency.

Looking at the slopes of the regression lines assessing the association between the Stroop scores and the eye movement latency, none of them are significant, which is also the case when considering Group E alone. Thus, although they are both similarly affected by age, they do not affect each other.

## 4. Discussion

The first major finding of the study is evidence of aging’s effects on hearing, inhibition and the latency of both saccades and vergence eye movements. Although prior studies exist considering one aspect or the other, to our knowledge, no studies investigated all of these aspects together on the same population. The second major finding is evidence of a correlation between speech recognition in silence and the latency of saccades regardless of age. These results will be discussed below.

### 4.1. A Normal Aging Population

As this study assesses the relationship between hearing, eye movement latency and Stroop scores for the aging population, it is important to put the aging effects found here in perspective with literature.

Concerning the hearing tests, our results show a physiologic degradation of all of the parameters of hearing capacities (pure-tone audiometry, and speech recognition in silence and in noise), and the percentage of participants who presented a hearing deficit was in the expected rates. The study of Lin et al. [3] found a hearing loss prevalence of 63% for US adults above 70 years of age, with a hearing loss definition as the PTA of the better ear being superior to 25 dB HL. By taking the same definition of hearing loss, the current results find a prevalence of 56% if we consider the subgroup composed only of participants aged 70 years and older. These two results can be considered consistent. The current study population was mainly composed of women (74% for Group E), and the prevalence of hearing loss for women is less than that for men [50]. Indeed, above 70 years of age, with the same definition of hearing loss as Lin et al., the subgroup composed of women has a prevalence of 50%, while the subgroup composed of men has a prevalence of 80%. As the number of male participants is limited, the total prevalence is lower than that reported by Lion et al.

Concerning the eye movement latency, the results of the current study also show a degradation of the latency for all of the eye movements measured (divergence, convergence, left saccade and right saccade). For saccade, these results are in line with the literature [26,27,28,29,30]. To our knowledge, this is the first study examining the aging effect on vergence latency movements in a relatively large population. The two prior studies including measures of vergence were limited to 30 patients [31,32]. The results found in the current study confirm an increase of vergence latency with age.

Concerning the Stroop scores, the results show a progressive deterioration with age of Stroop_I and Stroop_I/D for the whole population (Group E + Group Y) and for Group E alone. The Stroop_I scores are in line with the literature [37,38,39,40]; nevertheless, studies on the aging of Stroop_I/D are scarce and contradictory. As explained in the methods, Stroop_I/D (the time for the interference condition divided by the time for dot condition) allows us to be more specific regarding the inhibitory functions than Stroop_I (the time for the interference condition), which is more influenced by general age-related slowing. The study of Troyer et al. [41] on 272 participants (from 18 to 84 years) found an increase of the Stroop_I/D score with age, suggesting a loss of inhibition capacities. However, the studies of Bayard et al. [37] and Graf et al. [40], respectively on 244 participants (mean age 65.8 ± 10.7) and 129 participants (from 65 to 95 years) didn’t show a significant correlation between this ratio score and age, suggesting that the increase in time spent for Stroop tasks with age is only due to the general slowing, with inhibitory and attentional capacities remaining consistent. Therefore, even if the population sample of the current study is smaller than that of prior studies, the outcomes are in accordance with the study of Troyer, which used the English version of Stroop Victoria. Interestingly, the results in the current study for aging for Stroop_I/D are contradictory with those of the study of Bayard et al. (2011), which also used the French Stroop Victoria, for a population with the same age range.

To sum up, the results are globally consistent with the literature, showing a deterioration of hearing, latency and inhibitory capacity with age, suggesting that the aging effects described and the interactions described below may arise from physiological mechanisms. Moreover, all of the participants were autonomous or still-active professionals, and were carefully prescreened to exclude individuals with any type of pathology (neurological, psychiatric, no medication, no ocular diseases, with normal binocular vision except for a few surgeries for cataracts, no vertigo and equilibrium disorders).

Now, we will discuss the possible implications of the study’s major findings.

### 4.2. Improving Clinical Tools for Presbycusis Diagnosis

Presbycusis, and more generally hearing issues, are mostly determined in the clinic using three hearing tests: pure-tone audiometry thresholds, speech audiometry in silence and speech audiometry in noise. Although pure-tone audiometry thresholds have been the gold standard for the measurement of hearing impairment for roughly 100 years, it has become more obvious in the last few years that they can miss specific hearing issues, which are called “hidden hearing loss” [51]. This kind of hearing loss compromises the sound processing above the detection thresholds, and often translates into people with normal audiometric thresholds reporting difficulties in understanding speech in a complicated environment [52]. Aging is related to these “hidden hearing losses”, as speech intelligibility in background noise declines with age even when there is no significant increase in audiometric thresholds [53,54]. Some psychophysical and electrophysiological studies confirm this phenomenon by showing that temporal deficits appear with age, independently from an increase in the audiometric threshold [55,56,57].

Thus, presbycusis can degrade the audibility (which is assessed by pure-tone audiometry) and sound processing (which is essential for speech comprehension) in independent ways, and speech audiometry in silence and in noise is now systematically assessed in hearing evaluation. It is noteworthy that, in France, recent regulation enables the reimbursement of hearing aids by social security in the presence of an abnormality of either PTA or speech recognition in silence or noise.

In the current study, PTA and SRT50 are strongly correlated (*r* = 0.86, *p* = 0), but this is largely due to the method used to measure speech comprehension ability in silence. Indeed, PTA measures audibility (the minimal intensity required to detect a sound), and SRT50 also strongly depends on it. SRT50 only measure one characteristic of speech comprehension: the minimal intensity required from a word to be approximately understood (50% comprehension). For example, a participant with a bad PTA will also have a bad SRT50 because he wouldn’t even detect the words with low intensity. Even given this, there is still around 25% of the variability of the SRT50 that the PTA doesn’t explain. The results in Figure 4A notably show a consequent variability of SRT50 scores for the PTA between 10 and 20 dB HL. Therefore, it is interesting and justified to use speech-in-silence audiometry as a second criterion for hearing characterization, even for a normal hearing person according to the WHO criteria (PTA ≤ 20 dB HL).

The smaller correlation between PTA and SNR50 than between PTA and SRT50 is also expected. Indeed, inversely to SRT50, the SNR50 is not directly related to audibility: it doesn’t assess the minimal intensity required from a word to be understood in noise; rather, it assesses the extent to which increasing the background noise intensity will deteriorate the comprehension of a word which would be perfectly understood in silence. The weak correlation (albeit significant) between SNR50 and PTA is in line with the literature: pure-tone audiometry is not a good predictor of speech-in-noise audiometry. In this way, people who normally detect sound can have abnormal difficulties in understanding in noisy environments [58,59,60,61]. Therefore, these results confirm the importance of considering the speech-in-noise audiometry regarding the speech-in-silence audiometry and the pure-tone audiometry thresholds in order to obtain a more precise evaluation of hearing capacities.

However, even these three measures together are far from representing our total hearing abilities. PTA, SRT50 and SNR50 do not fully represent audibility and speech comprehension abilities in silence and in noise. For example, PTA is limited to a few frequencies (250, 500, 750, 1000, 2000, 3000 and 4000 Hz). SRT50 represents only one characteristic of the speech comprehension ability, i.e., the minimum intensity required to obtain 50% comprehension in silence, for a specific setup, and when the subject is fully focused on that task. Even if other variables than SRT5 can be extracted from speech audiometry in silence, this test does not assess, for example, the listening effort required or the mental replacement. The same problems apply to SNR50. Moreover, it is even more difficult for speech audiometry in noise to provide an accurate reflection of the real situations, as it is very difficult to recreate a noisy environment in a sound booth cabin, and there are many different noisy situations. In that respect, research on additional tools for hearing issue diagnosis is of high interest.

The major finding of the current study, i.e., the significant link between SRT50 and the saccade latency independently from age, suggests a promising way forward for further research. As PTA and SRT50 are highly correlated, it is interesting to note that saccade latency is significantly associated with SRT50 but not with PTA. The difference between them is that SRT50 not only relies on audibility but also on speech processing.

Thus, we hypothesize that saccade latency reflects, in some way, the speech processing abilities, and may become a diagnostic tool for it. Further studies on that topic would be interesting if they took other setups allowing a better focus of the speech processing abilities to the detriment of audibility. However, apart from such potential interests to develop other clinical tools of presbycusis diagnosis, the question is which mechanisms underlie such an association between saccade latency and speech processing.

### 4.3. Cognitive Mechanisms Implied in Hearing Processing

As mentioned in the introduction, the activated mechanisms during eye movement latency—i.e., attention, motor preparation and decision—are highly related to cognitive executive functions, and are subtended by a wide cortical visual parietal frontal network. This is confirmed by literature that found an association between saccade latency and cognitive health [33,34,35]. The fact that saccade latency is significantly linked to SRT50 but not to PTA suggests that saccade latency reflects, to some extent, the speech processing ability. While audibility arises from peripheral auditory processing, speech perception is a complex process between perceptual, sensory and cognitive abilities, and thus arises from a more central process than from sound detection alone. Thus, some cognitive processes of speech comprehension could be related to those of saccade latency. The results presented here tend to show, for the first time, that the cognition involved during speech comprehension processing shares characteristics with that involved during saccade latency.

None of the Stroop scores assessed here were significantly related to hearing abilities, whether this is PTA, SRT50 or SNR50, suggesting that selective attention is not relevant for hearing processing. However, there is already existing literature suggesting that inhibition and selective attentional capacities (measured with Stroop tests) have an important role in speech comprehension [49,62]. The assumptions are that, independently of sensory impairment and general slowing, poor selective attention will increase (i) the susceptibility to be distracted by a background noise during a speech-in-noise listening [62], and (ii) the difficulty to successfully recognize an item among other items with similar acoustics features [49]. However, these studies, described later, used different paradigms than the current one, which could explain the inconsistent results.

Concerning the first assumption, the background noise used in the study of Janse et al. [62] was distracting speech understandable by the participant. By contrast, the noise used in the current study for the speech audiometry in noise was an incomprehensible babble noise. Therefore, it is unlikely that a participant with poor attention capacities will be distracted by that noise. The second assumption was based on the NAM (neighborhood activation model) of lexical discrimination [63]. This model represents a classification of words in our mental lexicon into similar neighborhoods. The word recognition process is performed by comparing lexical items heard with the different words in a subgroup of neighborhood words, and active inhibition is the mechanism allowing the brain to easily delete the neighborhood words with irrelevant lexical contents in order to target the good word. The studies of Sommers et al. [64,65] found that the age-related decrease of selective attentional functions appears to play a significant role in the increasing difficulty with the recognition of complex words (words with a high density of lexical neighborhoods) for older adults. However, the important discrepancy between our study and those of Sommers et al. is that they assessed selective attention capacities related to auditory tasks, while our study assessed them via a visual task. Therefore, it appears that the importance of inhibition and selective attention for comprehension according to the NAM model depend on the sensorial modality: inhibition measured with an auditory task will be linked to speech comprehension, and inhibition measured with a visual task will be linked with the comprehension if the vision is also implied. This is consistent with other studies of Sommers et al. [66] and Helfer and Freyman [67]. The study of Sommers found a significant relationship between visual Stroop scores and comprehension in noise, but for an audiovisual speech (speech with lip reading). Helfer and Freyman failed to show a link between speech comprehension without visual cues and a visual Stroop test.

To sum up, saccade latency seems to be a promising new approach to estimate and measure the cognitive process involved in speech processing.

These mechanisms didn’t appear to be related to the selective attention involved in a visual task. However, this last assumption required further research. It would be interesting to use an inhibition test implying auditory modality, as was shown in the studies of Sommers et al., or to use the Visual Stroop test with other paradigms.

## 5. Conclusions

This is a pioneering study on the relationship between the normal aging of eye movement latency, hearing and cognition capacities. The auditory and cognitive consequences of presbycusis are of high importance, and current hearing tests sometimes may not be sufficient to accurately evaluate hearing capacities [51,52,58,59,60,61]. Thus, there is a need to develop knowledge on the mechanism binding hearing and cognition, and to bring new helpful measures for hearing loss diagnosis. We hope the current study will stimulate further research on the relationship between speech processing abilities and eye movement latency.

## 6. Patents

**REMOBI:** US885 1669, WO2011073288, AIDEAL: PCT/EP2021/062224 7 May 2021.

## Figures and Tables

**Table 1:** Correlations and regression lines between the Stroop scores and age.

	Stroop ~ Age					
	Group Y + Group E			Group E		
Stroop Variable	Intercept	a	cor	Intercept	a	cor
Stroop_D	10,407	0,033 **	0,283	13,171	−0,008	−0,031
Stroop_W	10,526	0,081 ***	0,537	11,363	0,068	0,207
Stroop_I	12,96	0,191 ***	0,581	12,899	0,192 *	0,265
Stroop_W/D	1,049	0,003 ***	0,383	0,905	0,006 *	0,259
Stroop_I/D	1,332	0,011 ***	0,467	1,101	0,015 *	0,255

“***” for a *p* inferior to 0.001, “**” for a *p* between 0.001 and 0.01, “*” for a *p* between 0.05 and 0.01.

**Table 2:** Multiple regressions: hearing results as a function of eye movement latency and age.

	Hearing ~ Latency + Age Group Y and Group E															
	Divergence				Convergence				Left Saccade				Right Saccade			
		a	StdError	*t* Value		a	StdError	*t* Value		a	StdError	*t* Value		a	StdError	*t* Value
PTA	Latency	−0,013	0,014	−0,894	Latency	−0,005	0,014	−0,327	Latency	0,015	0,016	0,976	Latency	0,02	0,016	1,268
	Age	0,367 ***	0,044	8,346	Age	0,353 ***	0,041	8,639	Age	0,329 ***	0,045	7,366	Age	0,322 ***	0,045	7,173
SRT50	Latency	−0,012	0,014	−0,838	Latency	0,003	0,014	0,178	Latency	0,033 *	0,015	2,175	Latency	0,043 **	0,015	2,85
	Age	0,279 ***	0,044	6,394	Age	0,261 ***	0,04	6,44	Age	0,218 ***	0,043	5,045	Age	0,203 ***	0,043	4,754
SNR50	Latency	0,008	0,006	1,218	Latency	0,005	0,006	0,832	Latency	0,009	0,007	1,343	Latency	0,005	0,008	0,685
	Age	0,03	0,02	1,504	Age	0,039 *	0,017	2,294	Age	0,032.	0,018	1,743	Age	0,037.	0,019	1,914

“***” for a *p* inferior to 0.001, “**” for a *p* between 0.001 and 0.01, “*” for a *p* between 0.05 and 0.01, “.” for a *p* between 0.1 and 0.05.

**Table 3:** Multiple regressions: hearing results as a function of Stroop scores and age.

	Hearing ~ Stroop + Age Group E and Group Y							
	Stroop_I				Stroop_I/D			
		a	StdError	*t* Value		a	StdError	*t* Value
PTA	*Stroop*	−0,067	0,138	−0,484	*Stroop*	0,821	1,81	0,453
	*Age*	0,361 ***	0,05	7,213	*Age*	0,337 ***	0,046	7,347
SRT50	*Stroop*	0,089	0,136	0,651	*Stroop*	2,211	1,774	1,246
	*Age*	0,249 ***	0,049	5,033	*Age*	0,241 ***	0,045	5,368
SNR50	*Stroop*	−0,072	0,055	−1,309	*Stroop*	−0,178	0,756	−0,235
	*Age*	0,063 **	0,02	3,168	*Age*	0,051 **	0,019	2,734

“***” for a *p* inferior to 0.001, “**” for a *p* between 0.001 and 0.01.

**Table 4:** Multiple regressions: eye movement latency as a function of Stroop scores and age.

	Latency ~ Stroop + Age Group Y and Group E							
	Stroop_I				Stroop_I/D			
		a	StdError	*t* Value		a	StdError	*t* Value
Divergence	*Stroop*	−1,112	1,054	−1,054	*Stroop*	−7,423	13,518	−0,549
	*Age*	1,487 ***	0,347	4,29	*Age*	1,357 ***	0,32	4,233
Convergence	*Stroop*	1,569	1,144	1,372	*Stroop*	17,257	14,637	1,179
	*Age*	0,359	0,376	0,956	*Age*	0,468	0,347	1,348
Left Saccade	*Stroop*	−0,522	0,913	−0,572	*Stroop*	9,344	11,637	0,803
	*Age*	1,478 ***	0,3	4,925	*Age*	1,275 ***	0,276	4,622
Right Saccade	*Stroop*	−0,163	0,933	−0,174	*Stroop*	13,132	11,839	1,109
	*Age*	1,418 ***	0,307	4,621	*Age*	1,241 ***	0,281	4,423

“***” for a *p* inferior to 0.001.

## Data Availability

Data are available upon reasonable request.
